# Text Messaging Interventions for Unhealthy Alcohol Use in Emergency Departments: Mixed Methods Assessment of Implementation Barriers and Facilitators

**DOI:** 10.2196/65187

**Published:** 2025-03-03

**Authors:** Megan O'Grady, Laura Harrison, Adekemi Suleiman, Morica Hutchison, Nancy Kwon, Frederick Muench, Sandeep Kapoor

**Affiliations:** 1 Department of Public Health Sciences, University of Connecticut School of Medicine, 263 Farmington Avenue, Farmington, CT, 06032, United States, 1 860 679 5483; 2 Northwell Health, New Hyde Park, NY, United States; 3 Zucker School of Medicine at Hofstra/Northwell, Hempstead, NY, United States; 4 Partnership to End Addiction, New York, NY, United States

**Keywords:** unhealthy alcohol use, text messaging intervention, emergency department, barriers and facilitators, implementation, alcohol use, unhealthy, mixed methods assessment, mixed methods, assessment, facilitators, alcohol, health care, scalable supports, text messaging, patient outcomes, health system, electronic health record, EHR, alcohol screening, acceptability, feasibility, survey, health-related goals

## Abstract

**Background:**

Many patients with unhealthy alcohol use (UAU) access health care in emergency departments (EDs). Scalable supports, such as SMS text messaging interventions, are acceptable and feasible to enhance care delivery for many health issues, including substance use. Further, SMS text messaging interventions have been shown to improve patient outcomes related to alcohol consumption (eg, reduced consumption compared to no intervention, basic health information, or drink tracking), but they are rarely offered in clinical settings.

**Objective:**

This paper describes a mixed methods study using the Integrated Promoting Action on Research Implementation in Health Services (i-PARIHS) framework. The goal of this study was to use a stakeholder-engaged mixed methods design to assess barriers and facilitators to the implementation of SMS text messaging interventions for UAU in EDs with a focus on the recipient’s characteristics, the innovation’s degree of fit within the existing practice, and the unique nature of the inner and outer context.

**Methods:**

This study was conducted in a large health system in the northeastern United States. We examined electronic health record data on alcohol screening in 17 EDs; surveyed 26 ED physician chairpersons on implementation feasibility, acceptability, and appropriateness; and interviewed 18 ED staff and 21 patients to understand barriers and facilitators to implementation. Interviews were analyzed according to the i-PARIHS framework to assess recipient characteristics, innovation degree of fit, and inner and outer context.

**Results:**

Electronic health record data revealed high variability in alcohol screening completion (mean 73%, range 35%‐93%), indicating potential issues in identifying patients eligible to offer the intervention. The 26 ED chair surveys revealed a relatively high level of implementation confidence (mean 4, SD 0.81), acceptability (mean 4, SD 0.71), and appropriateness (mean 3.75, SD 0.69) regarding the UAU SMS text messaging intervention; feasibility (mean 3.5, SD 0.55) had the lowest mean, indicating concerns about integrating the text intervention in the busy ED workflow. Staff were concerned about staff buy-in and adding additional discussion points to already overwhelmed patients during their ED visit but saw the need for additional low-threshold services for UAU. Patients were interested in the intervention to address drinking and health-related goals.

**Conclusions:**

ED visits involving UAU have increased in the United States. The results of this formative study on barriers and facilitators to the implementation of UAU SMS text messaging interventions in EDs indicate both promise and caution. In general, we found that staff viewed offering such interventions as appropriate and acceptable; however, there were concerns with feasibility (eg, low alcohol risk screening rates). Patients also generally viewed the SMS text messaging intervention positively, with limited drawbacks (eg, slight concerns about having time to read messages). The results provide information that can be used to develop implementation strategies that can be tested in future studies.

## Introduction

Unhealthy alcohol use (UAU) costs the United States nearly US $250 billion per year [1], and alcohol-related deaths among those aged 16 years and older recently increased by 25% [2]. Emergency departments (EDs), where alcohol-related visits are rising [3-5], are sometimes the only health care touchpoint for patients with UAU, making it a promising point of intervention [6,7]. SMS text messages are one of the most ubiquitous and salient modes of mobile health interventions. They use brief, supportive messages to promote behavior change without significant user burden [8-10] and are acceptable and feasible to enhance care delivery for many health issues, ranging from high blood pressure to substance use [9,11-16]. A meta-analysis and study indicated that alcohol-targeted texting interventions reduced alcohol consumption compared with no intervention, basic health information, or drink tracking [17,18]. Notably, UAU text interventions for ED and trauma patients are acceptable and effective in reducing drinking [19-22]. However, there is limited uptake of text-messaging interventions in ED settings.

Reviews of substance use technology interventions have noted minimal focus on implementation strategies or outcomes; rather, most studies focus only on patient clinical outcomes [23,24]. Thus, prioritizing the examination of barriers and facilitators to understand ways to increase uptake is an important next step [25]. This paper describes a mixed methods study using the Integrated Promoting Action on Research Implementation in Health Services (i-PARIHS) framework, which posits optimal implementation occurs when interactive problem-solving promotes acceptance and use of a new innovation based on the recipient’s characteristics (eg, motivation), the innovation’s degree of fit within the existing practice (eg, usability), and the unique nature of the inner (eg, culture) and outer context (eg, mandates) [26,27]. The examination of barriers and facilitators to implementing UAU text interventions in EDs [26,28] was done by examining electronic health records (EHRs), surveys, and interview data from a large health system using i-PARIHS elements known to influence the uptake of new innovations.

## Methods

### Study Population

The study was conducted in 17 adult-serving EDs in the northeastern US urban and suburban locations; it is part of a larger project examining SMS text messaging interventions for UAU [29].

### Ethical Considerations

This study was approved by the institutional review board at the Feinstein Institutes for Medical Research (approval number 21-0756). All participants consented to study procedures using verbal consent processes and consent information sheets. All qualitative data were deidentified prior to analysis; survey data were anonymous. Patients received a US $15 gift card incentive; staff participants were not compensated. Generative artificial intelligence was not used in any part of the manuscript writing.

### Procedures and Materials

We used an explanatory, sequential mixed methods approach (quantitative → qualitative) [30]. Data were integrated at several points. Quantitative EHR and survey data classified sites on low or high implementation potential; classifications were used for data collection and site selection for qualitative interviews and analysis. Data were integrated during result interpretation. Reporting is in accordance with the COREQ (Consolidated Criteria for Reporting Qualitative Research) and CHERRIES (Checklist for Reporting Results of Internet e-Surveys) checklists for qualitative health research involving interviews and internet e-surveys.

### EHR Data

The 3-item AUDIT-C (Alcohol Use Disorders Identification Test-Consumption) [31] detects UAU (≥3 for women and ≥4 for men) and was built into all participating ED EHRs; it provided information on each ED’s capacity to address UAU (eg, low screening completion rates may indicate a lack of time). Data, extracted from 2021, included percentage of patients screened (number of AUDIT-C completions divided by the total patient census).

### Staff Surveys

ED physician chairpersons from the 17 participating EDs received an email from the study team inviting them to complete a 13-item web-based survey of three validated 4-item scales assessing conceptually distinct, but interrelated constructs often used in formative research to examine implementation barriers: feasibility (eg, “A text messaging intervention for UAU seems possible in my ED”), acceptability (eg, “I would welcome our clinical team offering a text messaging intervention for UAU to patients during their ED visit”), and appropriateness (eg, “A text messaging intervention for UAU seems fitting for patients in my ED”) [32]. An additional confidence item (“I am confident that our clinical ED team would offer a text messaging intervention for UAU to patients during their ED visit”) was included. All items were rated on a 5-point scale (1=completely disagree to 5=completely agree). To complete site classification, we calculated each site’s survey totals (range 4‐65; higher values indicate positive implementation outcomes). All invited EDs participated; in cases where both primary and secondary chairs completed the survey, scores were averaged.

### Implementation Potential Classification

We classified low versus high implementation potential based on AUDIT-C completion rates and survey scores (low: ≤median on both measures; high: >median on both measures), resulting in 5 low sites and 4 high. Sites with mixed classifications were not recruited. We considered several factors (eg, census and location) to select the 4 final EDs for interviews (2 low and 2 high) using stratified purposeful sampling [33,34].

### Staff Interviews

In the 4 final EDs, chairpersons were interviewed and then each identified staff. They were invited via emails from study staff, resulting in 18 (82%) out of 22 invited interviewies. Interviews lasted ~30 minutes, were conducted over Zoom, recorded, and cleaned for analysis. Interview guides covered views on alcohol screening, SMS text messaging interventions and how to best offer them, barriers and facilitators to intervention implementation, and general practice change.

### Patient Interviews

Patients were recruited from 6 EDs; sites were selected based on location and census to gain perspectives from different populations and ED sizes (eg, race or ethnicity and urban or suburban). Patients were approached during their ED visit if they screened positive on the AUDIT-C. Of the 30 patients invited, 21 (70%) were interviewed. Interviews lasted ~20 minutes and were recorded and transcribed. The interview guide was adapted from previous ED studies (eg, mobile device use and interest in receiving supportive messages on UAU reduction) [35,36].

### Qualitative Analysis

We used rapid qualitative analysis [37] to analyze interview data following procedures previously outlined [37,38]. Two researchers (MO and AS) summarized 3 transcripts from patient and staff interviews to ensure consistent summaries. One researcher (AS) completed the remaining summaries with the second researcher (MO) reviewing for accuracy and alignment; discrepancies were resolved via discussion. For patients, we created comparison groups between AUDIT-C scores indicative of moderate to high risk (score 4‐7) versus severe risk (score 8‐12) to examine response differences; for staff, we created comparison groups by low versus high implementation potential sites. See for demographic information for chair surveys (n=26), staff (n=18), and patient interviews (n=21).

## Results

### EHR and Chair Survey

Average AUDIT-C completion rates (73%) were relatively high but ranged greatly (35%‐93%). Survey implementation scores indicated a relatively high level of confidence, acceptability, and appropriateness regarding the UAU texting intervention; feasibility had the lowest mean, indicating logistical implementation concerns. See Multimedia Appendix 2 for absolute values on AUDIT-C completion rates and implementation scores.

### Staff Interviews

Table 1 illustrates implementation barriers and facilitators. In comparing staff in low (n=7) versus high (n=11) implementation sites, low sites more frequently indicated addressing alcohol use was not a high priority. All staff discussed challenges to include information about the intervention in discharge papers, noting the paperwork is bulky and patients rarely read it. Facilitators were similar across high and low sites. Low sites discussed the difficulty of making changes, and that change takes time and requires strong justification. When asked about recent practice changes, low sites had fewer examples of what worked well and more frequently mentioned low staff morale, high turnover, and burnout.

**Table 1.** ED^a^ staff qualitative interview barrier and facilitator themes from rapid analysis in accordance with i-PARIHS^b^ domains.

Overall theme		Subtheme	Exemplar quote	i-PARIHS domain
Implementation barriers				
	Screening views	There is not 100% buy-in from staff to do alcohol risk screening (eg, feeling that patients are not truthful on screenings, that the threshold is too low for “positive,” that there is screening fatigue, or that patients may be surprised by being asked).	“...We try to have as good of a completion as possible...there isn’t a one hundred percent buy in from the staff when it comes to it...a lack of an education, because the staff is in our population as well...when you work in an emergency room, and you see a lot of crazy things, and you deal with a lot of crazy health issues, somebody comes in for one thing, you’re not necessarily too concerned about doing a screening kind of unrelated to the main diagnosis. But I think that is part is the main reason why completion rates aren’t one hundred percent...”	Context/inner (culture/climate)
	Screening process	Physicians or other providers generally do not view alcohol screening results.	“It’s kind of cumbersome, unless it’s actually pertinent to the case...We’re juggling so much. It’s a little too much for us to handle.”	Context/inner (culture/climate)
	Need for services	There was a focus mostly on the need for services for those with more severe problems who are frequent visitors to the ED.	“I think that it could be very helpful for a certain set of patients...the patients that keep coming back that we’ve tried to help...And yet, a week later they back.”	Recipients (motivation/beliefs)
	ED as an appropriate setting for addressing alcohol use	The ED is viewed as a difficult environment to address problems (lack of privacy and acute health problems) and have conversations beyond screening, doing more falls to low mixed priority due to staff workload/burden unless the patient is there for an alcohol issue.	“...I think it really depends on if the patient is there, specifically for the alcohol issue...In addition to the medical aspect, we try to make it a priority. But if a patient is identified in triage,...needing an SBIRT consult and they’re not there for then it’s not a priority it just becomes another thing in the chart that you know we just don’t have time to address, unfortunately.”	Context/inner (culture/climate)
	SMS text messaging intervention usefulness for ED patients	The text intervention was viewed as potentially less useful for patients with severe alcohol problems, less motivated to change their drinking, or certain populations (eg, older adults and low mobile phone users).	“To be honest, I don’t think it would be that effective to the majority, the patients that we see like the so-called frequent flyers, or the one-time binge drinker kind of deal. They’re not going to be interested in that...”	Innovation (degree of fit)
	Reasons patients may or may not accept the text intervention	Some patients may not have mobile phones/texting plans or may have lower motivation to change, which may deter from accepting a text intervention.	“...Some of them don’t have cell phones or some of them have like prepaid cell phones...I honestly don’t think it would be helpful.”	Innovation (degree of fit)
	Offering the text intervention to patients	Patients are overwhelmed during the ED visit and receive a lot of discharge instructions, so text intervention information could get lost.	“...whereas the doctor might put it on the discharge paper...who reads them? They’re twelve pages. It’s going in the garbage. No one is really you know paying attention to it.”	Innovation (degree of fit)
	Barriers to implementing a text intervention in the ED	It was expressed that physicians and nurses do not have the time to discuss alcohol use with patients, and that there are too many screens already being done, and the EHR^c^ is already full of things to click.	“...It would be tough to operationalize that in a busy...any ED but...to have that as another initiative for our PAs, attendings for cases that are not necessarily for that, may be difficult to operationalize. It’s not impossible I just don’t know if it would be feasible in every single case.”	Recipients (time, resources, support)
	Staff training needs	Staff are already overwhelmed with training; it would be difficult to add more training modules for a new intervention.	“...We get so many emails and have so many required modules that we need to watch that I think that those would probably not be the most effective way...”	Recipients (time, resources, support)
	General ease of making practice changes in ED	For something new to be implemented, staff must see value for the patient, and it must fit into the workflow without adding a burden.	“...when the staff buy into it and understand why we’re doing it for the patient. I think that helps. They need to understand why we’re implementing change.”	Context/inner (innovation/change)
	Reflections on recent practice changes	It was perceived that change takes a long time based on recent experiences in implementing new practices.	“...I think it could be challenging to make changes. Especially if it requires like extra work. I think for people, it takes time or some of them just will never do it that they’re just not complying with the changes. But I think it does take time and people to get used to the idea.”	Context/inner (innovation/change)
	Culture of innovation	Staff morale and burnout are still recovering from COVID and some long-time staff are not as open to change; this creates some ambivalence about making changes.	“...burnt out, probably from the most part, I think most staff is burnt still from Covid, and is still in a recovery phase in general, because volume is kind of coming back, everybody is a little deconditioned in terms of volume, because we had very low levels, and we had high levels. So I think there’s a level of burnout out there that we’re constantly battling that’s probably the biggest challenge that we’re having at the moment.”	Context/inner outer (culture/climate)
Implementation facilitators				
	Views on alcohol screening	The ED is a good place for screening because alcohol-related health issues in the ED are common, and it is beneficial information for the clinical team because the problem may not be identified or visible otherwise; it helps to normalize and destigmatize the issue of substance use.	“...I think it’s a pretty good place. Um, Ah to identify um people who have alcohol abuse because, like yeah, like, I said, we do have like a nice, decent amount of people that do come in...”	Context/inner (culture/climate)
	Views on the alcohol screening process	Most patients are being screened. AUDIT^d^ screening tool is built into EHR, and most patients are initially screened by triage/primary nursing and followed up via social work or screening, brief intervention, and referral to treatment (SBIRT^e^) health coaches.	“AUDIT screening tool is built into EMR, and most patients are initially screened by triage/primary nursing and followed up via social work or SBIRT.”	Context/inner (culture/climate)
	The need for services for people with alcohol problems	It was viewed that the prevalence of those with alcohol problems in the ED is high; they see people across the continuum of use.	“...I see people on two ends of the spectrum right. Either they’re completely intoxicated, and they’re over utilizing the system for a bed to sleep, or they’re coming in from like a bar or a nightclub overly intoxicated. Or I see the other end of this spectrum, which is somebody that’s in severe withdrawal, delirium tremens. I’ve seen people that need to be admitted to the ICU setting or a hospital monitored bed because that’s the normal vital signs risk procedure...Then you also see the other complications of alcohol abuse, such as like pancreatitis, for example, That’s...the majority of the cases that I interact with.”	Recipients (motivation/beliefs)
	ED as an appropriate setting for addressing alcohol use	The ED is a good place to identify problems and get the conversation started. The ED is a key access point that sees a high volume of people.	“I think it’s a good place to identify...I think there are resources around that we can pull from. But again, if there’s not a coach or somebody here, we struggle with where to refer people to.”	Context/inner (culture/climate)
	SMS text messaging intervention usefulness for ED patients	It was viewed that there was no downside to offering the text intervention, even with a potentially small/incremental benefit, and patients may be more inclined because it is something they can engage with after an ED visit and because it is automated.	“I think it would be helpful for a patient that would be willing and wanting to need services...it really just offers another resource. I think nowadays one of the many issues that are occurring is that patients don’t have access to resources. So I think any step that we can do to help provide other resources to patients that are in need, would be helpful. Something in real time or something where they don’t have to wait on the phone don’t have to dial number can just kind of use their phone and text would be definitely helpful. I think it would be good.”	Innovation (degree of fit)
	Reasons patients may or may not accept the text intervention	The text intervention could be introduced to patients in a way that describes how it could be beneficial while ensuring privacy/confidentiality.	“...just letting them know that it’s confidential, that it’s helpful for them. They might, maybe someone of a clinical nature could tell them that they think it’s important that they address this with someone, with a professional.”	Innovation (degree of fit)
	Offering the text intervention to patients	Ways to facilitate offering the text intervention could be pagers/flyers with strong marketing, including it in discharge instructions, and using a team approach to offering. Materials plus a human touch would be beneficial.	“...The other way to do this is a passive way in terms of flyers...somebody could just opt in reading material while they are there...then in terms of explaining it is probably just a script so you give them a flyer and you can train probably a lot of support staff to say I know this is not the reason for your visit today we are working on this initiative, We give this to everybody...”	Innovation (degree of fit)
	Barriers to implementing a text intervention in the ED	Tapping into already existing workforce members (eg, social work, SBIRT, and referral coordinators) could be useful for offering the text intervention.	“...I find that nurses have more time to talk to patients, but I think it would have to be a team approach coming from the attending and working with the nurses...I think it would have to really be a team effort on everybody’s part.”	Recipients (time, resources, support)
	Barriers to implementing a text intervention in the ED	It could be useful to plug into already operating workflows or offer passive ways of offering the text intervention (eg, QR code).	“...like QR Codes for people to open up YouTube videos as distraction videos...But I think that places like the bathroom walls and provide to our codes for them to scan phone numbers and then champions. And as for health coaches and our social workers, and our case managers are often utilized to help with, uh disposition of those patients, right?”	Context/inner (innovation/change)
	Staff training needs	Brief training using familiar modalities would help with educating team members about the text intervention (eg, learning management system modules, morning huddles, Microsoft PowerPoint slides with all relevant information about the text program, and morning brief).	“...PowerPoint that you could always like reference or something that they could go back on. Um. I know a lot of the times like providers kind of say like, Oh, social work could do this...Some type of education um would be good, I think, including maybe, examples of what the text messages are, and then examples of like, If they did reach out to the program, what are they able to actually help with...”	Recipients (time, resources, support)
	General ease of making practice changes in ED	The ED is a setting used to change and is very adaptable. It values flexibility and change and is nimble.	“...I hate to say it depends on the change right so, yeah, but I think I think to answer your question a little easier our department is open to change pretty regularly we change things, most emergency departments do but we’re pretty aggressive about being flexible, we pride ourselves sort of on that...”	Context/inner (innovation/change)
	Reflections on recent practice changes	EDs have a lot of recent experiences with change and methods to disseminate information and gain feedback and to have consistency in communication.	“...the changes with COVID testing and requirements and admission process...but I think what went well, people appreciate consistency, I think in communication, so I think what I appreciate now is that everyone knows, where to look for updates and things like that...”	Context/inner (innovation/change)
	Culture of innovation	EDs are overall welcoming of innovation and embrace a culture of change; leadership and a cohesive team are important in change/innovation.	“There has been a lot of change here. It’s always changing. They’ve received multiple certifications. (redacted). Um, They’ve made new rooms. They’ve gone through a renovation. They’ve gone through new leadership...”	Context/inner outer (culture/climate)

a ED: emergency department.

b i-PARIHS: Integrated Promoting Action on Research Implementation in Health Services.

c EHR: electronic health record.

d AUDIT: Alcohol Use Disorders Identification Test.

e SBIRT: Screening, Brief Intervention, and Referral to Treatment.

### Patient Interviews

Patient results indicated strong interest and potential benefit from a UAU text intervention with limited concerns (Table 2). When comparing patients who screened moderate to high (n=6) versus severe (n=15) on the AUDIT-C, only patients at moderate to high risk said they had a goal to use the intervention to prevent future heavy drinking, while both groups mentioned abstinence and moderation. Perceived benefits also varied. Patients at severe risk thought the program could help stop the progression of alcohol use disorder symptoms, change their lifestyle, and be a distraction from drinking; patients at moderate to high risk thought the program could improve their job roles and family relationships, and provide information about drinking and change their thoughts about drinking. Both groups thought the intervention could provide support and improve their health and had similar content preferences. Patients at severe risk additionally mentioned using the text intervention to track drinking, set and complete goals, engage family support, avoid people who trigger their use, and reduce heavy drinking during drinking episodes.

**Table 2.** ED^a^ patient qualitative interview themes from rapid analysis in accordance with i-PARIHS^b^ domains.

Overall theme	Subtheme	Exemplar quote	i-PARIHS domain
Patient phone use patterns	Most patients had mobile phones and used them for a variety of purposes, including frequent texting It was rare that patients used their mobile phones for health or to manage their alcohol use	N/A	Recipients Time, resources, and support Motivations, values, and beliefs
Interest in a text intervention for alcohol use	Most patients were interested in receiving the text intervention Patients were interested in different goals (eg, to prevent future heavy drinking, reduce current drinking, or stop drinking completely) Patients were interested in a variety of reasons (eg, improve health and relationships and change drinking cognitions)	“Yeah. Maybe for myself if, if, you know, in the future, I decide to drink – I start drinkin’ more. When I see myself drinkin’ more, I wanna know information. Maybe I could prevent myself from, you know, gettin’ into alcoholism or somethin’ like that.” [Participant 27] “Personally, maybe it might change my mind on why I’m drinking at that moment. Or if I’m feeling low, maybe it’d inspire me not to drink as much. Maybe those texts’ll be useful.” [Participant 17]	Innovation (degree of fit)
Content	Patients were interested in a variety of content (eg, quotes, uplifting, inspirational, or humorous messages, memes, images, resources, videos, and links with information) Patient preference on the desired frequency of texts ranged from 3 times per week to several times per day	“I like resources. Resources as in like what you should do besides picking up a drink.” [Participant 13]	Innovation (degree of fit)
Offer preferences	Most patients had no preference of who (eg, nurse or doctor) should offer the intervention, but it was more important that it be a personal conversation showing concern and empathy Most patients had no preference for whether they would prefer the intervention to be offered through the health system versus a third party	“Um, maybe towards the end and I’m, um, gettin’ discharged or somethin,’ like, when I feel better.” [Participant 27]	Innovation (degree of fit)
Concerns	Patients did not generally cite concerns related to privacy or cost; concern was that the program does not send too many messages, that they would not be able to read messages immediately, or that messages would be too long	“So if I can’t get to the message right away, I won’t. But usually when I open the messages up, I do take concern in what’s being said or written.” [Participant 17]	Innovation (degree of fit)
Motivation to change	Most participants had at least some motivation to change their drinking (eg, quit or cut down) and mentioned several reasons (eg, family and job)	“I’m at a 10 where I wanna change, Um, I have tried. I cut back, um, ‘cause I used to drink like every day and I cut back a lot, so…looking to cut it back even further to, and just stop drinking alcohol.” [Participant 25]	Recipients (motivations, values, beliefs)
Benefits to enrolling	Patients saw a number of benefits to enrolling (eg, help with mental and physical health, receiving information about negative effects of alcohol, having help in limiting alcohol use, or prompting in the moment to think differently about drinking)	“It just healthy to stop. Make your body feel better, more healthy.” [Participant 26]	Innovation

a ED: emergency department.

b i-PARIHS: Integrated Promoting Action on Research Implementation in Health Services.

## Discussion

The results of this formative study on barriers and facilitators to implementing UAU texting interventions in EDs indicate both promise and caution. Quantitative data suggested concerns with feasibility (eg, low alcohol risk screening rates and feasibility scores). Qualitative results confirmed and extended quantitative results; providers viewed offering the intervention as appropriate and acceptable, especially to address the high volume of alcohol-related visits and support patients longer term; patients viewed the intervention as a positive way to meet drinking goals and saw several health benefits; they saw limited drawbacks (eg, slight concerns about time to read messages).

Providers felt there was no downside to offering the intervention even if there was incremental patient benefit. However, differences were noted among low and high implementation potential sites: variable screening rates and lower ratings on feasibility (eg, competing priorities, staff burnout, and turnover), indicating potential additional support strategies needed to create staff buy-in and sustain intervention engagement in low-potential sites. Larger EDs with higher patient census’ may have unique implementation barriers, as they tended to have lower alcohol risk screening completion rates that would impact their ability to identify those who would benefit from text interventions. Other studies examining provider acceptance, barriers, and facilitators to implementing technology-based interventions in their EDs (not focused on alcohol use, however) revealed similar findings: high ratings of usage and value, but lower rates of actual use due to insufficient training, lack of awareness of the program, and technical factors (eg, compatibility) [39,40]. Future studies should examine ED characteristics that may impact implementation and design and test specific strategies to address these characteristics.

Patients of varying alcohol use risk severity indicated potential differences in drinking goals: those with higher severity may be less likely to have moderation goals and more likely to have abstinence goals. Results may reflect differences in how drinking has differently impacted health, relationships, functioning, and experience of alcohol use disorder symptoms among those with lower or higher severity. Patients’ reasons for using a text program for UAU in our study were similar to that of another study examining patients’ reasons for attending a residential program for alcohol use, including health, lifestyle, and sobriety goals [41]. Future studies of SMS text messaging programs for UAU should take into consideration different patient goals and experiences and how this could impact implementation strategies to engage patients. Designing programs that can address both moderate and more severe patients’ needs and uses is important; the ability of the program to tailor to the needs and preferences of individual patients should be inherent in the design.

This study had limitations, including being conducted in one health system, which may affect generalizability, and classifying implementation potential using the 5-point Likert scale, which was meaningful in identifying potential implementation issues, but may not indicate practically meaningful differences.

In conclusion, SMS text messaging interventions for alcohol use have the potential to offer ED staff another tool to assist patients with meeting alcohol-related health goals. Patients welcome these interventions as a way to receive support and information. However, EDs will have varying levels of motivation and readiness to implement these driven by barriers related to feasibility. Findings may be useful for the development and testing of implementation strategies in larger cluster-randomized trials, an important area for mobile behavioral health [42]. Automating enrollment (eg, QR codes and text invites), capitalizing on existing workflows, and engaging ED leadership may be fruitful implementation strategies to test in future ED implementation research.

## Supplementary material

10.2196/65187 Multimedia Appendix 1 Demographics for chair surveys (n=26), staff interviews (n=18), and patient interviews (n=21).

10.2196/65187 Multimedia Appendix 2 ED EHR alcohol screening data and feasibility, appropriateness, acceptability, and confidence of implementing alcohol SMS text messaging intervention in the ED in 17 sites. ED: emergency department; EHR: electronic health record.

10.2196/65187 Checklist 1 COREQ (Consolidated Criteria for Reporting Qualitative Research) checklist.

10.2196/65187 Checklist 2 CHERRIES (Checklist 2 for Reporting Results of Internet e-Surveys) checklist.

## Abbreviations

AUDIT-C: Alcohol Use Disorders Identification Test-Consumption
CHERRIES: Checklist for Reporting Results of Internet e-Surveys
COREQ: Consolidated Criteria for Reporting Qualitative Research
ED: emergency department
EHR: electronic health record
i-PARIHS: Integrated Promoting Action on Research Implementation in Health Services
UAU: unhealthy alcohol use
