# Supplementary material for: Text Messaging Interventions for Unhealthy Alcohol Use in Emergency Departments: Mixed Methods Assessment of Implementation Barriers and Facilitators
Source: JMIR Form Res. 2025 Mar 3;9:e65187. doi: 10.2196/65187 (PMC11892540; doi:10.2196/65187)
Supplement: Multimedia Appendix 1 [file formative-v9-e65187-s001.docx]

Multimedia Appendix 1. Demographics for chair surveys (n = 26), staff interviews (n = 18), and patient interviews (n = 21)

| Characteristic | Chair Surveys, N (%) or mean | Staff Interviews, N (%) or mean | Patient Interviews, N (%) or mean |
| --- | --- | --- | --- |
| **Age**, mean ± SD (Min-Max) | 46.2 ± 7.8  (35-67) | 42.4 ±11.5  (28-67) | 41.3 ± 14.6  (23-72) |
| **Gender** | | | |
| Male | 19 (73.1) | 8 (44.4) | 14 (66.7) |
| Female | 6 (23.1) | 10 (55.6) | 6 (28.6) |
| Gender queer/Gender non-conforming/neither exclusively male nor female |  |  | 1 (4.8) |
| Declined to answer | 1 (3.8) |  |  |
| **Race** | | | |
| White | 19 (73.1) | 12 (66.7) | 6 (28.6) |
| Black or African American | 2 (7.7) | 2 (11.1) | 8 (38.1) |
| Asian | 3 (11.5) | 3 (16.7) | 1 (4.8) |
| American Indian or Alaska Native |  |  | 1 (4.8) |
| Native Hawaiian or Pacific Islander |  |  | 1 (4.8) |
| Other | 1 (3.8) | 1 (5.6) | 4 (19.0) |
| Decline to answer | 1 (3.8) |  |  |
| **Ethnicity** | | | |
| Hispanic | 1 (3.8) | 1 (5.6) | 9 (42.9) |
| Non-Hispanic | 24 (92.3) | 17 (94.4) | 12 (57.1) |
| Declined to answer | 1 (3.8) |  |  |
| **Educational Level** | | | |
| Bachelor's Degree |  |  | 2 (9.5) |
| Associate Degree or Some College |  |  | 8 (38.1) |
| Graduated from high school or have GED or equivalent |  |  | 8 (38.1) |
| Some high school, no degree |  |  | 2 (9.5) |
| Declined to answer |  |  | 1 (4.8) |
| **Current position/title in the ED** | | | |
| Physician Chair (incl. Vice, Assoc, Interim) | 21 (80.7) | 6 (33.3) |  |
| Other physician leadership (site director, executive director, medical director) | 5 (19.2) | 2 (11.1) |  |
| Attending Physician |  | 1 (5.6) |  |
| Physician Assistant |  | 1 (5.6) |  |
| Nursing Director/Manager |  | 4 (22.2) |  |
| Other (referral coordinator, social worker, health coach, admin manager) |  | 4 (22.2) |  |
