# Supplementary material for: Text Messaging Interventions for Unhealthy Alcohol Use in Emergency Departments: Mixed Methods Assessment of Implementation Barriers and Facilitators
Source: JMIR Form Res. 2025 Mar 3;9:e65187. doi: 10.2196/65187 (PMC11892540; doi:10.2196/65187)
Supplement: Multimedia Appendix 2 [file formative-v9-e65187-s002.docx]

Table 2. ED EHR alcohol screening data and feasibility, appropriateness, acceptability, confidence of implementing alcohol text messaging intervention in the ED in 17 sites

| ED | Annual Census | % AUDIT-C screening completion | Appropriateness (total) | Feasibility (total) | Acceptability (total) | Confidence (score) | Total of four scales |
| --- | --- | --- | --- | --- | --- | --- | --- |
| All (avg) | 43470 | 73 | 15 | 14 | 16 | 4 | 49 |
| 1^a^ | 17782 | 88 | 20 | 16 | 20 | 4 | 60 |
| 2 | 46639 | 78 | 16 | 12 | 15.5 | 3.5 | 47 |
| 3 | 38526 | 48 | 16 | 14 | 18 | 4 | 52 |
| 4^a^ | 57680 | 67 | 10 | 12.5 | 11 | 2.5 | 36 |
| 5 | 48861 | 74 | 16 | 14 | 20 | 5 | 55 |
| 6^a^ | 89364 | 63 | 12 | 15.5 | 16.5 | 3.5 | 47.5 |
| 7 | 37664 | 86 | 8.5 | 11 | 13 | 3 | 35.5 |
| 8 | 33300 | 93 | 16 | 15 | 17 | 3 | 51 |
| 9 | 70233 | 70 | 15 | 14.7 | 15 | 4.5 | 49.2 |
| 10 | 26124 | 87 | 12 | 12 | 12 | 2 | 38 |
| 11 | 33575 | 45 | 17 | 16 | 17 | 4 | 54 |
| 12 | 26338 | 87 | 16 | 15.5 | 16 | 3.5 | 51 |
| 13 | 22488 | 90 | 12.5 | 12 | 12.5 | 4 | 42 |
| 14 | 72792 | 60 | 16 | 16 | 19.5 | 4 | 55.5 |
| 15^a^ | 26785 | 87 | 16 | 16 | 19.5 | 4 | 55.5 |
| 16 | 78530 | 35 | 14 | 14 | 14 | 2 | 44 |
| 17 | 12305 | 90 | 16 | 16 | 16 | 4 | 52 |

Note. ^a^Indicates site included in the staff interviews.
