# Supplementary material for: Text Messaging Interventions for Unhealthy Alcohol Use in Emergency Departments: Mixed Methods Assessment of Implementation Barriers and Facilitators
Source: JMIR Form Res. 2025 Mar 3;9:e65187. doi: 10.2196/65187 (PMC11892540; doi:10.2196/65187)
Supplement: Checklist 1 [file formative-v9-e65187-s003.docx]

Consolidated criteria for reporting qualitative studies (COREQ): 32-item checklist

| No Item, Guide questions/description  **Domain 1: Research team and reflexivity**  **Personal Characteristics**  1. Interviewer/facilitator Which author/s conducted the interview or focus group?  2. Credentials What were the researcher’s credentials? E.g. PhD, MD  3. Occupation What was their occupation at the time of the study?  4. Gender Was the researcher male or female?  5. Experience and training What experience or training did the researcher have? Relationship with participants  6. Relationship established Was a relationship established prior to study commencement?  7. Participant knowledge of the interviewer What did the participants know about the researcher? e.g. personal goals, reasons for doing the research  8. Interviewer characteristics What characteristics were reported about the interviewer/facilitator? e.g. Bias, assumptions, reasons and interests in the research topic  **Domain 2: study design**  **Theoretical framework**  9. Methodological orientation and Theory What methodological orientation was stated to underpin the study? e.g. grounded theory, discourse analysis, ethnography, phenomenology, content analysis  **Participant selection**  10. Sampling How were participants selected? e.g. purposive, convenience, consecutive, snowball  11. Method of approach How were participants approached? e.g. face-to-face, telephone, mail, email  12. Sample size How many participants were in the study?  13. Non-participation How many people refused to participate or dropped out? Reasons?  **Setting**  14. Setting of data collection Where was the data collected? e.g. home, clinic, workplace  15. Presence of non-participants Was anyone else present besides the participants and researchers?  16. Description of sample What are the important characteristics of the sample? e.g. demographic data, date  **Data collection**  17. Interview guide Were questions, prompts, guides provided by the authors? Was it pilot tested?  18. Repeat interviews Were repeat interviews carried out? If yes, how many?  19. Audio/visual recording Did the research use audio or visual recording to collect the data?  20. Field notes Were field notes made during and/or after the interview or focus group?  21. Duration What was the duration of the interviews or focus group?  22. Data saturation Was data saturation discussed?  23. Transcripts returned Were transcripts returned to participants for comment and/or correction?  **Domain 3: analysis and findings**  **Data analysis**  24. Number of data coders How many data coders coded the data?  25. Description of the coding tree Did authors provide a description of the coding tree?  26. Derivation of themes Were themes identified in advance or derived from the data?  27. Software What software, if applicable, was used to manage the data?  28. Participant checking Did participants provide feedback on the findings?  Reporting  29. Quotations presented Were participant quotations presented to illustrate the themes / findings? Was each quotation identified? e.g. participant number  30. Data and findings consistent Was there consistency between the data presented and the findings?  31. Clarity of major themes Were major themes clearly presented in the findings?  32. Clarity of minor themes Is there a description of diverse cases or discussion of minor themes? | Y/N, page number |
| --- | --- |
|  |  |
|  | Y, 18 |
|  | Y, 1 |
|  | N |
|  | N |
|  | N |
|  |  |
|  | Y, 6 |
|  | Y, 7 |
|  |  |
|  | Y, 7 |
|  |  |
|  |  |
|  |  |
|  | Y, 5, 7 |
|  |  |
|  |  |
|  | Y, 7,8 |
|  | Y, 7,8 |
|  |  |
|  | Y, 7,8 |
|  | Y, 7,8 |
|  |  |
|  | Y, 7,8 |
|  | N |
|  | Y, 20 |
|  |  |
|  |  |
|  | Y, 7,8 |
|  | N/A |
|  | Y, 7,8 |
|  | Y,7,8 |
|  | Y, 7,8 |
|  | N |
|  | N |
|  |  |
|  |  |
|  | Y, 8,9 |
|  | N |
|  | Y,8,9,11,12 |
|  | N/A |
|  | N  Y, 22-31  Y  Y, 22-31  N/A |
