# Supplementary material for: Text Messaging Interventions for Unhealthy Alcohol Use in Emergency Departments: Mixed Methods Assessment of Implementation Barriers and Facilitators
Source: JMIR Form Res. 2025 Mar 3;9:e65187. doi: 10.2196/65187 (PMC11892540; doi:10.2196/65187)
Supplement: Checklist 2 [file formative-v9-e65187-s004.docx]

**Checklist for Reporting Results of Internet E-Surveys (CHERRIES)**

| ***Item Category*** | ***Checklist Item*** | ***Explanation*** |
| --- | --- | --- |
| **Design** | Describe survey design | Target population was ED Chairs, collected via convenience sampling of 17 EDs participating in the study. |
| **IRB approval and informed consent** | IRB Approval | Approved by the IRB at the Feinstein Institutes for Medical Research |
|  | Informed Consent | All participants consented to study procedures. |
|  | Data Protection | Basic demographics were collected, all data was stored on locked, university owned computers. Only study team members had access to data. |
| **Development and pre-testing** | Development and testing | Survey was developed by study team members to include demographics, demographic categories have been tested prior in the field. Implementation scales are validated measures. |
| **Recruitment process and description of the sample having access to the questionnaire** | Open survey versus closed survey | Open survey to all Chairs at participating EDs. |
|  | Contact mode | Initial contact was made via email, with a link to the online survey |
|  | Advertising the survey | Survey was announced via email by study team to participating EDs. |
| **Survey administration** | Web-E-mail | e-survey was sent out via email, where a link was provided for participants to manually enter answers. |
|  | Context | e-survey was on Qualtrics, a free, secure, web-based application to create and manage online forms, surveys, questionnaires and databases |
|  | Mandatory/voluntary | The survey was voluntary |
|  | Incentives | No incentives were offered |
|  | Time/Date | May 31^st^-June 9^th^ 2022 |
|  | Randomization of items or questionnaires | Items were not randomized. |
|  | Adaptive questioning | Adaptive questioning was not used. |
|  | Number of items | Number of items per page ranged from 7-14 |
|  | Number of screens (pages) | The survey was 2 pages. |
|  | Completeness check | All items were set to ‘required’, therefore no items were missing and completeness was 100%. All relevant items had a non-response option. |
|  | Review step | Respondents were able to review and change their answers. |
| **Response Rates** | Unique site visitor |  |
|  | View rate (ratio of unique survey visitors/unique site visitors) | This was a targeted survey sent to specifically designated chairs, of whom 100% participated. |
|  | Participation rate (Ratio of unique visitors who agreed to participate/unique first survey page visitors) | 100% |
|  | Completion rate (Ratio of users who finished the survey/users who agreed to participate) | 26/27 (96.3%) |
| **Preventing multiple entries from the same individual** | Cookies used | Cookies were not used for this survey. |
|  | IP check | We did not collect IP addresses as it is an identifier. |
|  | Log file analysis | N/A |
|  | Registration | This was a targeted survey with links sent to the designated chairpersons at participating hospitals. |
| **Analysis** | Handling of incomplete questionnaires | Only completed questionnaires were analyzed. |
|  | Questionnaires submitted with an atypical timestamp | No cut-off point was used, all surveys were completed in an acceptable amount of time ranging from 1:15 to 40:52, which is to be expected of active ED chairs during their shifts. |
|  | Statistical correction | Weighting of items or propensity scores were not used. |
